# Visualizing the knowledge structure and evolution of bioinformatics

**DOI:** 10.1186/s12859-022-04948-9

**Published:** 2022-09-30

**Authors:** Jiaqi Wang, Zeyu Li, Jiawan Zhang

**Affiliations:** College of Intelligence and Computing, Tianjin University, Tianjin, China

**Keywords:** Bioinformatics, Visualization, Knowledge structure, Text-mining

## Abstract

**Background:**

Bioinformatics has gained much attention as a fast growing interdisciplinary field. Several attempts have been conducted to explore the field of bioinformatics by bibliometric analysis, however, such works did not elucidate the role of visualization in analysis, nor focus on the relationship between sub-topics of bioinformatics.

**Results:**

First, the hotspot of bioinformatics has moderately shifted from traditional molecular biology to omics research, and the computational method has also shifted from mathematical model to data mining and machine learning. Second, DNA-related topics are bridge topics in bioinformatics research. These topics gradually connect various sub-topics that are relatively independent at first. Third, only a small part of topics we have obtained involves a number of computational methods, and the other topics focus more on biological aspects. Fourth, the proportion of computing-related topics hit a trough in the 1980s. During this period, the use of traditional calculation methods such as mathematical model declined in a large proportion while the new calculation methods such as machine learning have not been applied in a large scale. This proportion began to increase gradually after the 1990s. Fifth, although the proportion of computing-related topics is only slightly higher than the original, the connection between other topics and computing-related topics has become closer, which means the support of computational methods is becoming increasingly important for the research of bioinformatics.

**Conclusions:**

The results of our analysis imply that research on bioinformatics is becoming more diversified and the ranking of computational methods in bioinformatics research is also gradually improving.

## Background

Bioinformatics originated as a cross-disciplinary field because of the increasing need for computational solutions to research problems in biomedicine [1]. Since the field developed in leaps and bounds, researchers have shown an increasing interest in summing up the development and evolution of the entire discipline. However, the relationship and evolution of bioinformatics’s subtopics have unique characteristics due to its interdisciplinary nature. Most previous works failed to further explore this relationship and did not realize the great potential of visualization in exploring and displaying the evolution of this relationship.

### Traditional bibliometric analysis

Bibliometrics is the application of mathematics and statistical methods to evaluate the literature in different disciplines. Patra et al. [2] analyzed the growth of the scientific literature in bioinformatics. They applied Bradford’s law which estimates the exponentially diminishing returns of searching for references in science journals and Lotka’s law which is used to describe the frequency of publication by authors in any given field [3] to identify core journals and analyze author’s productivity pattern. W Glänzel et al. [4] proposed a novel subject-delineation strategy to retrieve of the core literature in bioinformatics. They then analyzed the core literature with bibliometric analysis tools such as co-author citation analysis, national publication activity, citation impact etc. Song et al. [5] conducted a bibliometric analysis of bioinformatics by extracting citation data from PubMed Central full-text. They focused on evaluating the productivity and influence of bioinformatics. Four measures were used to identify productivity: most productive authors, most productive countries, most productive organizations, and most popular subject terms. Research impact was analyzed based on the measures of most cited papers, most cited authors, emerging stars, and leading organizations.

### Text mining applied to bioinformatics bibliometrics

The development of text-mining techniques provides a new perspective for bibliometric analysis. Topic model is the most prevalent approach among techniques applied for bibliometric analysis. Latent Dirichlet Allocation(LDA) is one of the most popular models applied for bibliometric analysis. Song et al. [6] attempted to detect the knowledge structure of bioinformatics by applying LDA to a large set of bioinformatics full-text articles for topic model generation. Author-Conference-Topic (ACT) [7] model (an extension of LDA that can incorporate the paper, author, and conference into the topic distribution simultaneously.) was adopted by Heo et al. [8] to study the field of bioinformatics from the perspective of key phrases, authors, and journals. Heo analyzed the ACT Model results in each period to explore the development trend of bioinformatics

Traditional bibliometric analysis lacks thematic descriptions of bioinformatics subtopics, and the above-mentioned works filled that gap. However, LDA is a typical bag-of-words model that has two major weaknesses: it loses the ordering of the words and ignore their semantics. We choose another technical scheme: paragraph embedding [9], dimension reduction and clustering. Paragraph Embedding is an unsupervised framework that learns continuous distributed vector representations for pieces of texts which will take the ordering of words in to account. Its construction gives this algorithm the potential to overcome the weaknesses of bag-of-words models and allows us to capture more accurate features of documents to benefit document clustering. Thus, we can better map knowledge structure of bioinformatics.

### Visualization applied to bioinformatics bibliometrics

Scholarly data visualization enables scientists to have a better way to represent the structure of data sets and reveal hidden patterns in the data [10]. However, most previous studies did not realize the benefit of using visualization for exploring knowledge structure of bioinformatics. Visual mapping intuitively shows the overall knowledge structure, research framework, and development trends of a discipline that is very helpful for researchers to rapidly comprehend the overall research status and hotspots [11]. Although basic charts are widely used to display changes in the number of literatures [12] or the number of citation count [6], those charts lack of description of the overall knowledge structure and interaction to explore more information. Therefore, the advantages of visualization are not fully utilized. Another visualization tool used in bibliometrics analysis is network, which is commonly used for co-analysis, such as co-author analysis [13], co-citation analysis [6] and term co-occurrence analysis [14]. This type of network focuses on only one specific relationship, such as co-author focus on relationships between scholars. These diverse network structures are difficult to combine for comparative analysis. Furthermore, when dealing with big data set, the structure of graph may look like a hairball which is incomprehensible for analysts.

In this study, we fully utilized the ability of text mining technology to extract abstract paradigms from massive data and the ability of visualization to display complex information. First, we revealed the distribution of topics in two-dimensional document space by drawing scientific maps of bioinformatics, and then checked the evolution of topic relationships with time filters. Finally, to further understand the interdisciplinary nature of bioinformatics, we combined themeriver with words co-occurrence network to elaborate the evolution of computing-related topics.

Compared with previous works, present study has the following contributions: (1) A science map of bioinformatics was drawn to depict the intersection and evolution of sub-topics of bioinformatics. (2) The interdisciplinary nature of bioinformatics was explored emphatically analyzing the evolution of computing-related topics. (3) The validity and necessity of visualization in analysis were pointed out and proved.

## Results

In this section, we will introduce the final visualization results. The goals of our visualization are twofold: (1) intuitively show the change in the popularity of different topics over time; and (2) clearly demonstrate the relationship between topics and how this relationship changes over time

### Theme river

Goal 1 can be achieved by applying themeriver [15], where the color band represents clustering, and the width of the color band represents the number of articles in the clustering.

**Fig. 1:**
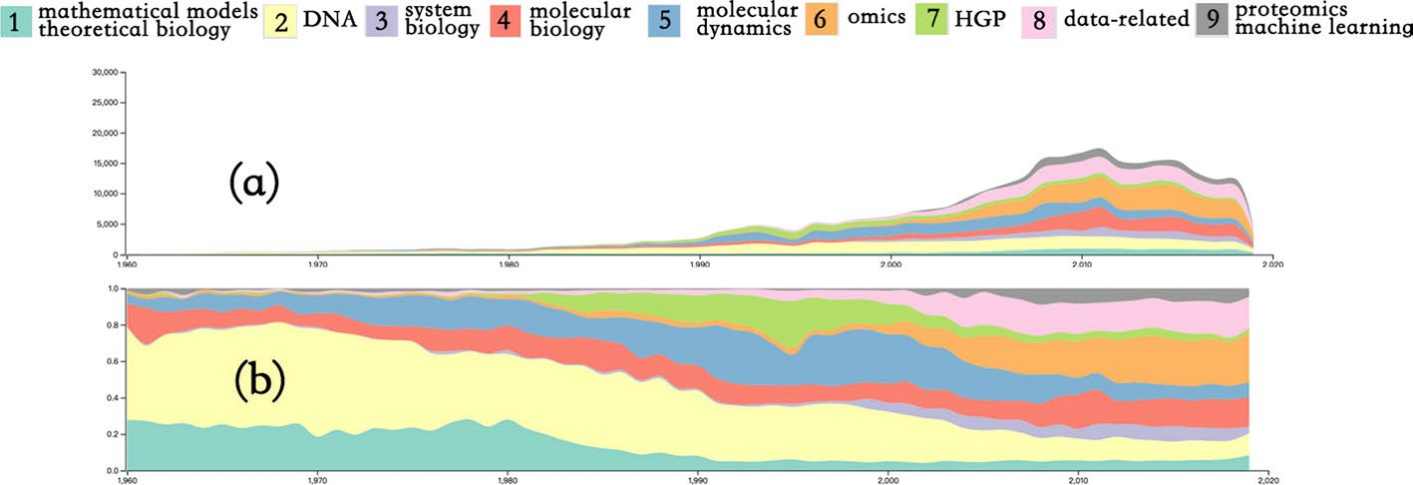
Themeriver of bioinformatics topics: Colors represent nine different topics; A focuses on quantitative changes; B reveals topic trends

As shown in Fig. 1, our theme river has two forms of expression. (a) The middle ordinate represents the actual number of documents, and the development of the origin of the overall discipline can be clearly seen. However, the number of documents is too far away from the origin stage because the development of the discipline has shown a violent growth trend in middle and late stages(From 72 papers in 1960 to more than 16,000 papers in 2010). Under such a quantitative gap, the category information from 1960 to 1990 is compressed by the scale, so that its internal category composition cannot be clearly seen in Fig. 1a. Therefore, we designed the second form of expression. The ordinate no longer represents the true value but the proportion of sub-topics. The value range is from 0 to 1. From the Figure, we can clearly see that the hot topics also change over time. Moreover, some topics are slowly falling out of sight over time.

### Science map

Science map originated from a traditional scientific notation called “knowledge tree” [16]. According to this tree structure, knowledge is divided into branches, which are then merged into main disciplines, and further divided into molecular disciplines and different specialties. However, with the continuous increase in the speed of knowledge dissemination, the exchanges between disciplines have become more intense, and more interdisciplinary disciplines have emerged. The tree structure has been difficult to meet this demand for discipline description. We need to use a more vivid and intuitive way to show the structure of bioinformatics and the impact of exchanges between different disciplines on bioinformatics’ structure.

**Fig. 2:**
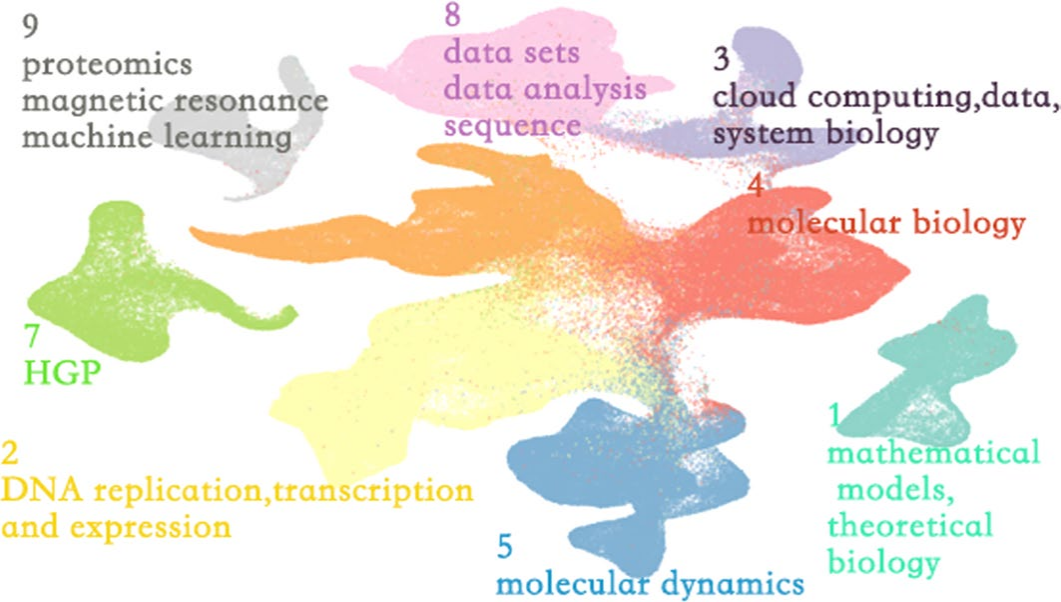
Scatterplot of bioinformatics

To show the relationship between bioinformatics papers in a two-dimensional space, we need to perform dimensionality reduction again on the document vectors obtained in the previous step. This time we still chose UMAP to reduce the vectors’ dimension to 2, which will enable us to project all vectors into two-dimensional space. We choose UMAP for two reasons: first, according to Espadoto et al. [17],UMAP performs well; second, it can support supervised/semi-supervised dimensionality reduction. To ensure that the data points that belong to two-dimensional space continue to maintain the structural information in the high-dimensional space, we first filtered the soft clustering results and selected those data labels whose category probability is greater than 0.9. We then used UMAP with those labels for semi-supervised dimensionality reduction. Finally, we projected the obtained vector into a two-dimensional space and presented it as a scatter plot shown in Fig. 2

**Fig. 3:**
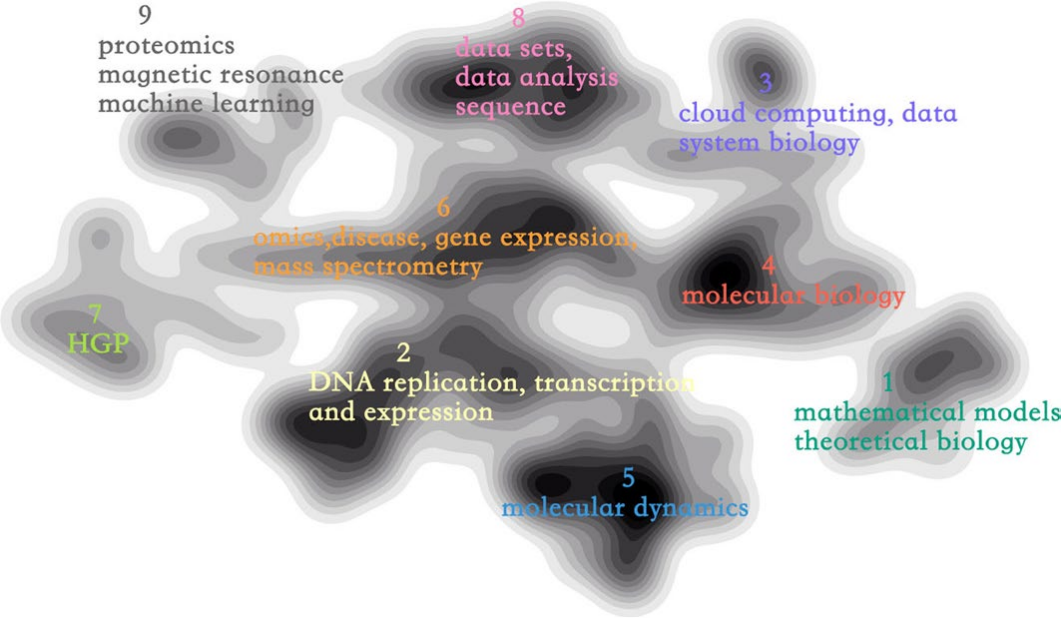
Knowledge structure of bioinformatics. Color opacity is proportional to the density of papers and the darker the color, the higher the density. Line spacing is inversely proportional to the density gradient. Descriptions for each cluster are summarized by TF-IDF

Each point represents a paper, and color codes the cluster it belongs to. However, when there is a larger number of points scatter plots may cause a misleading that the greater scope covered by an area, the more is the quantity of its papers. To eliminate this misleading and make the overall structure more visible, we implemented a contour line, where color depth is proportional to the density of papers, and line spacing is inversely proportional to the density gradient. The final science map of bioinformatics is shown in Fig. 3. We also supported interactive operation to help in-depth analysis, The high-frequency MESH phrases of the papers in the region of interest can be obtained by selecting the region.

## Discussion

In this section, we analyzed the results from three different perspectives: knowledge structure of bioinformatics, evolution of knowledge structure, and evolution of computing-related topics.

### Knowledge structure of bioinformatics

As shown in Fig. 1b, the popularity of topic 1(mathematical models, theoretical biology) and topic 2(DNA replication, transcription and expression), which are the initial main topics, have gradually decreased since 1980; meanwhile, topic 4(molecular biology) and topic 5(Molecular dynamics) remain relatively stable with limited change. Topic 6(genomics and proteomics) and Topic 8(data-related) have developed rapidly after 2000 and became the main topics of bioinformatics. Topic 3(system biology) and Topic 9(proteomics) were developed around 2000; although they did not receive too much attention, they have maintained a small but stable growth since 2000. The mode of Topic 7(HGP: The Human Genome Project) is somewhat special. This topic has been growing steadily since it appeared in 1980, reaching a peak in about 1995, but began to decrease greatly after 2000. This phenomenon can be well explained by combining the start and end time of the human genome project (1990-2003).

Although themeriver can describe the changes in research hotspots, it cannot sketch the relationship between topics and thus cannot accomplish our goal 2. Thus, we need a science to map the whole knowledge structure of bioinformatics. By analyzing Fig. 3, we obtain the following conclusions about the relationship between bioinformatics’ topics. (1) Topics 2, 4 and 6 are bridges connecting other topics. And they all deal with DNA indicating that DNA research is the ’skeleton’ of bioinformatics. (2) Among the topics distributed around the structure diagram, Topic 5 is related to Topic 2 and 4, while Topic 7 is mainly related to Topic 2 and 6. Topic 1 is relatively independent from other topics. (3) The three topics 3, 8 and 9 related to computing methods are all located at the top of the structural diagram. Topic 8 is at the center, which means that data are the core of computing-related topics of bioinformatics. (4) The closest non-computational topic to Topic 8 is Topic 6; hence, topic discussed in Topic 6 may use more methods of data science than other topics.

### Evolution of knowledge structure

With the continuous development of bioinformatics, the relationship between topics is also changing. From 1960 to 2019, the change in the number of papers on different topics is shown in Fig. 1a. We selected several time periods when the number of papers varied greatly.

**Fig. 4:**
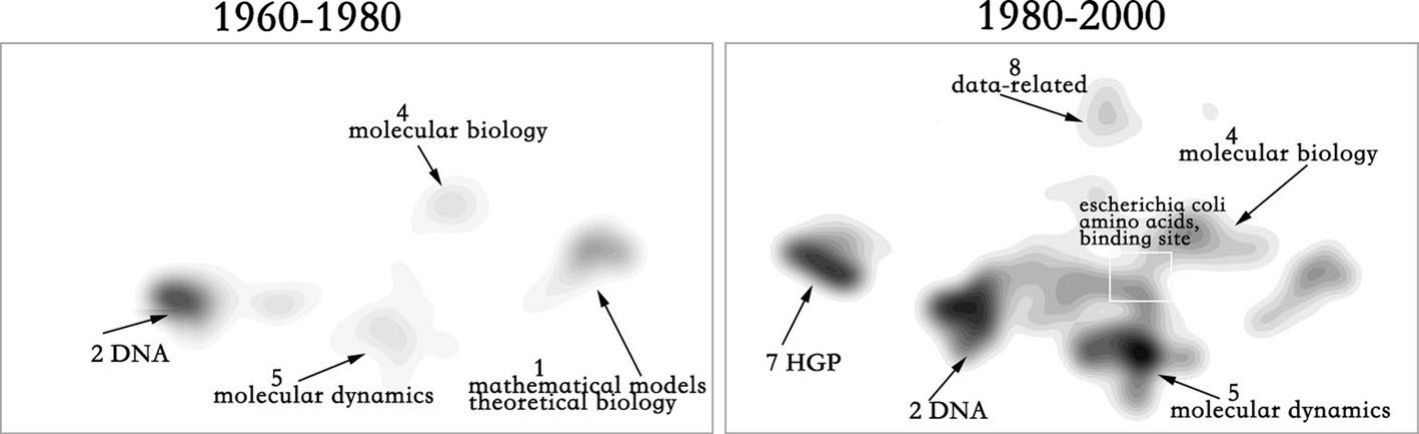
Evolution of bioinformatics from 1960 to 2000

At the beginning of the development of bioinformatics, the relationship between the main topics 1, 2, 4 and 5 was relatively weak. However, from 1980 to 2000, the 2,4,5 topics began to merge to a certain extent. We selected the fusion part interactively and examined at the high-frequency MESH phrases discussed in the merged papers. The top six phrases are: escherichia coli, amino acids, binding site, plasma membrane, binding sites, and signal transduction, which are all related to biological macromolecules (Fig. 4).

**Fig. 5:**
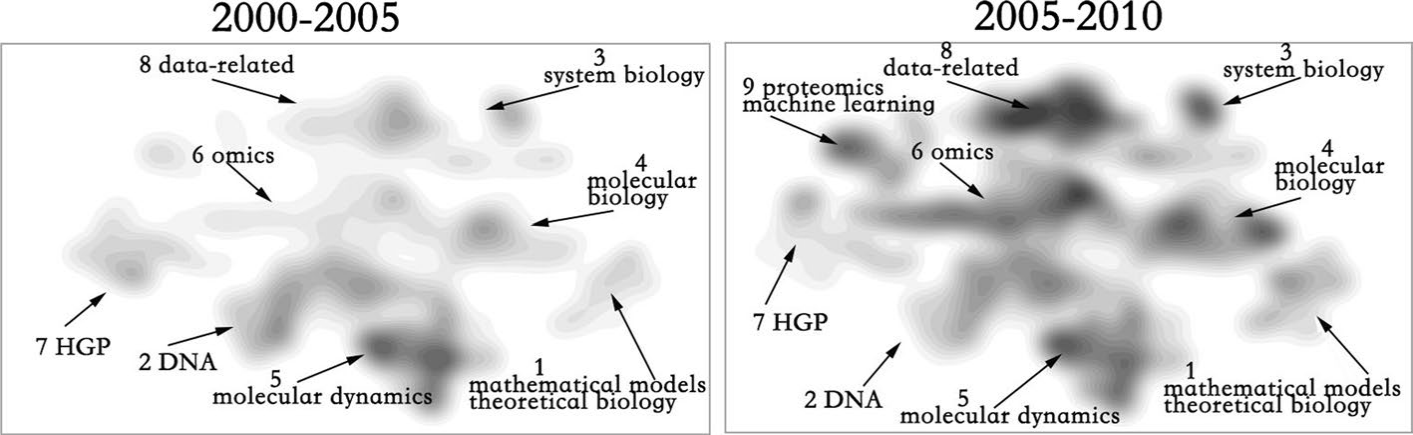
Evolution of bioinformatics from 2000 to 2010

From 2000 to 2005, the number of substructures of bioinformatics increased, but the upper and lower sides are relatively independent. Bridge topic 6(genomics and proteomics) has not become the main hot topic at that time, and has not produced more contact with Topic 3 and 8, but produced more fusion with topic 2, indicating that omics research began with DNA. The period from 2005 to 2010 is a period of vigorous development of bioinformatics. Topic 6, as a bridge topic and a hot topic, began to connect various parts (Fig. 5).

**Fig. 6:**
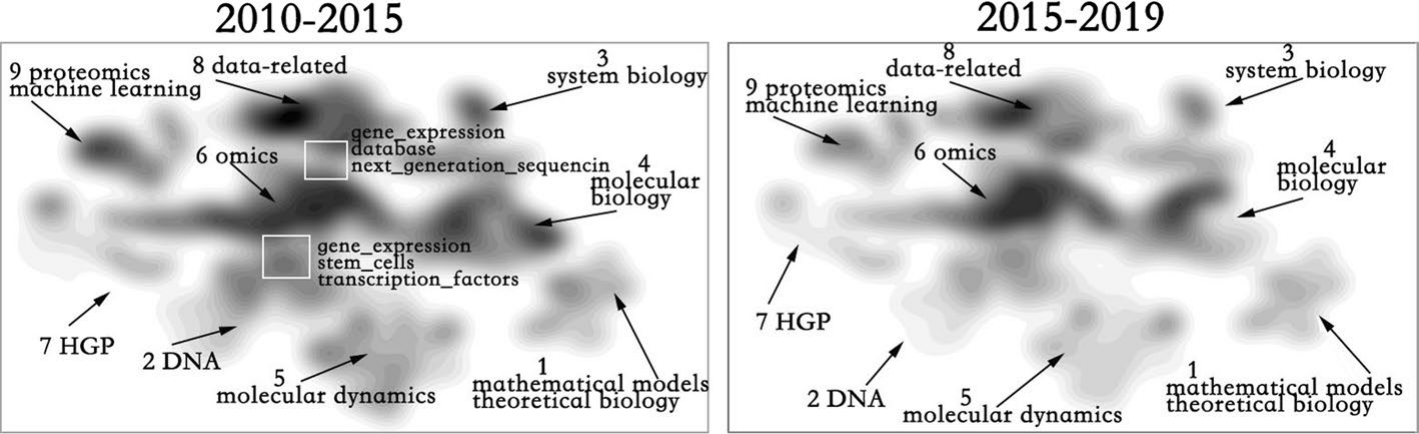
Evolution of bioinformatics from 2010 to 2019

After 2010, the hotspot began to move upward and the distance between Topic 6 and Topics 8,9 was shortened in this period. This finding indicates that omics research increasingly relied more on the support of computational methods (Fig. 6).

### Evolution of computing-related topics

Understanding the evolution of topics related to computation is crucial to understand the whole bioinformatics structure due to the special interdisciplinary nature of bioinformatics. We tried to answer two questions through visual analysis: (1) What is the proportion of computing-related topics in bioinformatics? Is this ratio stable? (2) What changes have taken place in the biological topics and computational methods involved in the evolution of these topics?

Goal 1 can be achieved by simply applying themeriver. We will mainly introduce the solution to Goal 2.

**Fig. 7:**
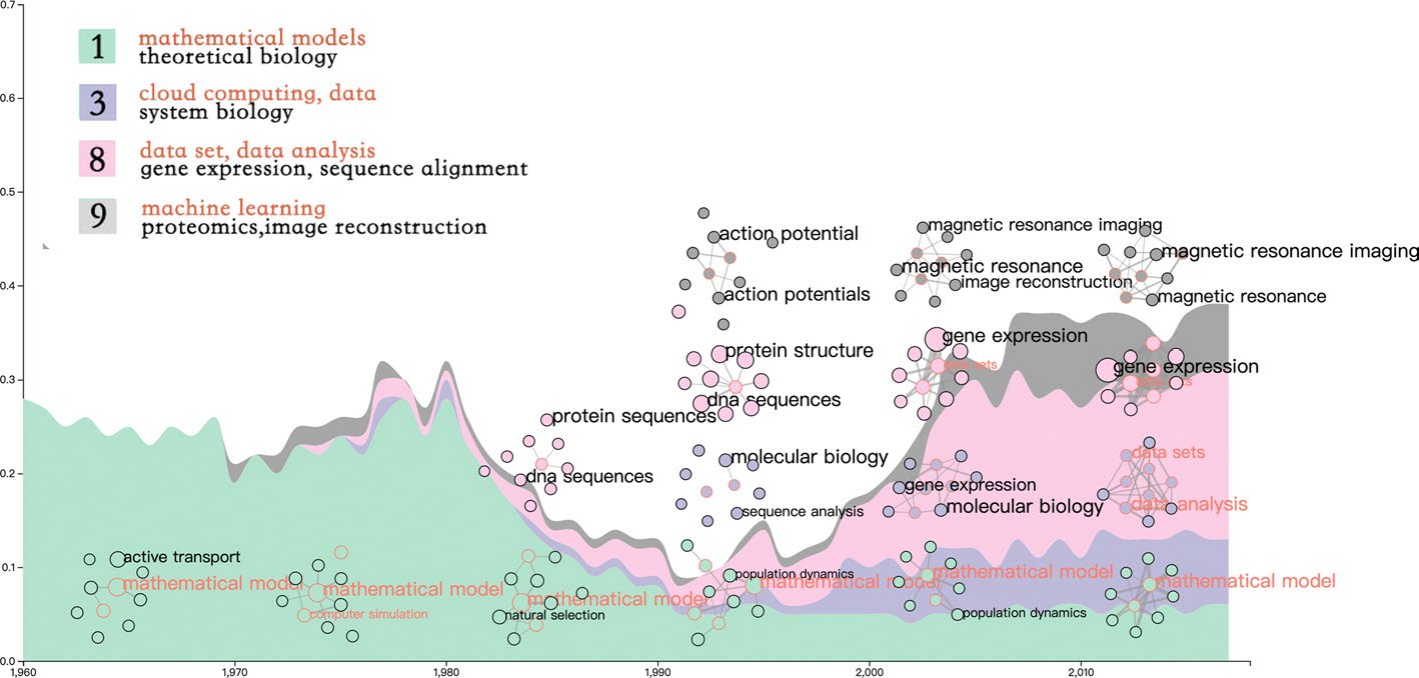
Evolution of computing-related topics. Themeriver shows the change in the proportion of papers and word co-occurrence network illustrates the change of topics in each period. Each circle represents a MESH phrase, and circle size represents the number of occurrences of the phrase

As shown in Fig. 7, themeriver shows the change of the proportion of four computing-related topics and the word co-occurrence network elaborates the change of topics in each period. By default, the word co-occurrence network of all topics is displayed. Click the category label to hide other categories and explore the selected topic in depth. MESH phrases with high frequency will be displayed by default. Moving the mouse over the circle will display its corresponding MESH phrase. The terms related to computation are represented by stroked circles and marked with orange.

The proportion of papers that related to computation evidently changes. The proportion was close to 0.3 in the early stage of the development of bioinformatics, began to decline in the early 1980s, and only accounted for about 0.1 of the total papers in the early 1990s. The proportion then began to rise again in the middle and late 1990s, surpassing the initial proportion and occupying nearly half of the bioinformatics topics after 2005. During such changes, an obvious pattern is observed: although the proportion of computing-related topics is only a little higher than the original, the internal structure of those topics has undergone earth-shaking changes.

Through further analysis of the co-occurrence word network, we conclude the following. (1) Prior to 1980, Topic 1 occupied an absolute dominant position. The computational methods involved were mainly mathematical models, and computer simulation appeared after 1970. The main biological topics discussed during this period include: active transport, cell, etc. (2) The period of 1990-2000 was a turning point for major changes in the internal structure of computing-related topics. The overall proportion began to rise, but the popularity of Topic 1 remained the same; and Topic 8 gradually replaced Topic 1 to occupy the dominant position. No new computational method was found in Topic 8, but the discussion on sequence increased significantly. (3)The period of 2000 - 2010 is an era of data and genes. The popularity of topics 3 and 8 continues to rise, but the content of the discussion has undergone great changes. The computational topics are all centered on data, data analysis, and the popularity of gene expression is the highest in biological topics while discussions on sequences began to decrease. Topic 9 has also seen a significant increase, and the computational methods involved are mainly related to data; however, biological topics focus on magnetic resonance and images. (4) In 2010 - 2019, the overall structure has not changed, and the proportion of each topic has remained relatively stable, however, the contents they discussed are different from those before. During this period, more computing methods are involved, such as machine learning, data mining, text mining, cloud computing and so on. Thus the interaction between computing methods and bioinformatics becomes increasingly closer and closer.

## Conclusion

As an interdisciplinary and fast-growing field of science, bibliometric analysis of bioinformatics has attracted the attention of many researchers. In this study, we collected 330192 bioinformatics papers and applied Doc2vec combined with clustering and dimension reduction technology to detect the knowledge structure of bioinformatics. And then we focus on the role of visual analysis in exploring this structure. Unlike previous works, we focus more on substructures’ relationship. The evolution process of computing-related topics was emphatically analyzed which is vital for understanding the interdisciplinary nature of bioinformatics. The results of our analysis imply that research on bioinformatics is becoming more diversified; the ranking of computational methods in bioinformatics research is also gradually improving. In the future, we plan to enrich and complete the knowledge structure diagram of bioinformatics by applying visualization to explore other aspects of bibliometric analysis, such as author analysis, organization analysis, citation analysis, etc.

## Methods

**Fig. 8:**
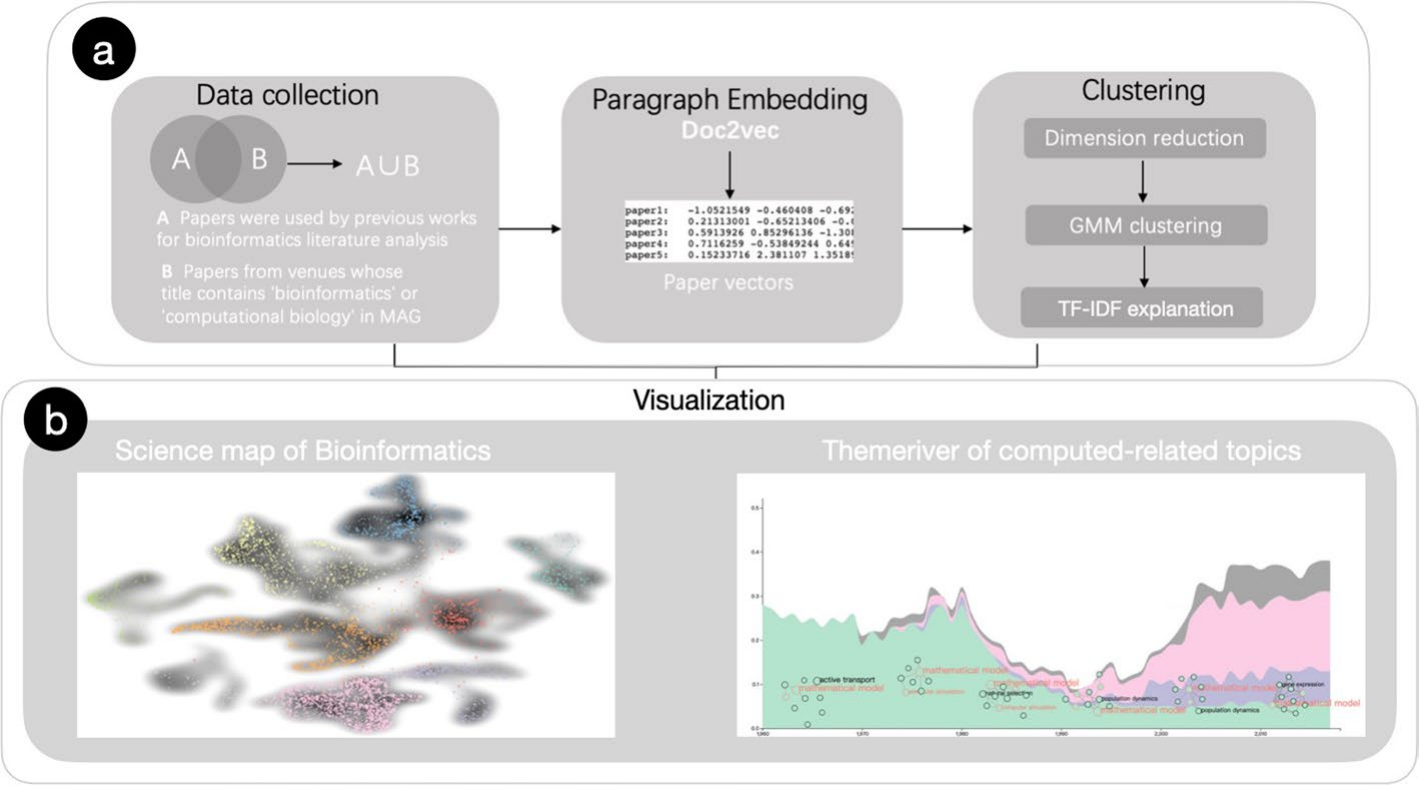
Pipeline of the proposed methods. **a** Shows the process of data collection and topic extraction. **b** Is our final visualization results. Science map shows the knowledge structure of bioinformatics and it can be filtered by time to show evolution.Themeriver shows the change in the proportion of papers and word cooccurrence network illustrates the change of topics in each period

In this section, we will introduce the overall procedure of the proposed approach for visualizing the knowledge structure of bioinformatics. Figure 8 shows the pipeline of our methods.

### Data collection and preprocessing

Data were obtained from Microsoft Academic Graph (MAG) [18]. MAG is a heterogeneous graph comprising more than 120 million publication entities and related authors, institutions, venues and fields of study. Papers in MAG that belongs to the union of two venue sets were analyzed: (1) 47 bioinformatics journals offered in Song [6], and 33 conferences used in Song [19]; (2) venues whose title contains ‘bioinformatics’ or ‘computational biology’ in MAG (“Appendix 1 and 2”). Corpus generation is mainly based on bioinformatics related venues. Papers of these venues have been strictly screened by review experts, thus they can better represent the knowledge structure of bioinformatics. This is also the reason why venue-filtered method is adopted in many previous works [5, 6, 13, 19].

A total of 330,192 papers were analyzed in the following discussion. We then extracted the title and abstract of these papers from the MAG to form a corpus. Detecting phrases in the corpus is crucial because the semantics of these phrases change once they are split into words. So after removing the stop words, we used phrases in Medical Subject Headings(MESH) to annotate phrases in our corpus. These phrases used to analyze bioinformatics literatures in many previous studies [2, 4, 6] to detect phrases in the corpus.

### Topic modeling

#### Paragraph embedding

Paragraph embedding is an unsupervised framework that learns continuous distributed vector representations for documents. Semantic similarity between documents can be obtained through calculating cosine similarity between vectors. Paragraph embedding is implemented in the gensim package (called doc2vec in gensim), in which the representation vector of each word in the corpus can be obtained at the same time as the document vector by setting parameter ‘dm’ to 1, which is used to calculate the semantic similarity between words. The selection of parameters is based on the best practice given by Lau [20]: window size is equals to 5 and epoch is equals to 600;

#### Clustering

To have a preliminary understanding of the knowledge structure of bioinformatics, we analyzed MESH term vectors first which is also better for subsequent document classification and interpretation. We used HDBSCAN [21], an outstanding soft clustering algorithm, to determine the clusters for MESH vectors. According to McInnes, HDBSCAN has the following characteristics compared to those parameter-sensitive algorithms: Performs DBSCAN over varying epsilon values and integrates the result to find a clustering that gives the best stability over epsilon. In practice this means that HDBSCAN returns a good clustering straight away with little or no parameter tuning. Phrases tend to be more specific than single word, so we chose to check on the top 10 most representative phrases in each category. Among nine clusters, only one cluster is related to computation, and the other clusters are all terms related to biology. Biology terms are mainly related to genes, proteins and sequences.

Clustering the document vectors can help us to obtain the topic distribution of bioinformatics and further understand the knowledge structure. Compared with LDA, our method can flexibly select the clustering method and the number of categories according to the clustering results after obtaining the vectors. After several rounds of attempts, we chose Gaussian Mixture Model (GMM). GMM is similar to K-Means, but it can be used as soft clustering, giving the probability of data points being assigned to a category.

To avoid high computational cost and remove noises of data, we used PCA to reduce the dimension of document vector from initial 300 to 50. To preserve data’s nonlinearity, we used the nonlinear dimensionality reduction algorithm UMAP [22] to further reduce the dimension of the document vector to 10. The parameters we used are as follows: n-neighbors is equal to 100 and dimension is equal to 10. These 10-dimensional vectors were used as the input of GMM. Basing on the clustering experience of MESH phrase with HDBSCAN, we selected the number of categories(the number of topics) to be 9. To explain the semantic meaning of each cluster, for each cluster, we used tf-idf algorithm to sort the words in the cluster, and selected the top 10 words as the descriptors of the cluster. Specifically, the tf-idf algorithm is as follows:

$$\begin{aligned} tf_idf_{i,j} = \frac{n_{i,j}}{\Sigma _{k} n_{k,j}} \times log\frac{|D|}{|{j:t_{i}\in d_{j}}|} \end{aligned}$$

where $n_{i}{j}$ is the count of word $t_{i}$ in cluster $d_{j}$ ,$\Sigma _{k}n_{k}{j}$ is the total count of all words in cluster $d_{j}$, |*D*| is the number of clusters and $|j:t_{i} \in d_{j}|$ is the number of clusters containing $t_{i}$.

Results are shown in Table 1. ‘compute’ means the words belongs to the category related to computation in the word vector classification, and ‘biology’ is the word in the eight other categories.

**Table 1:** Top 10 tf-idf words in each cluster

Topic1	Topic2	Topic3
**Compute**: mathematical- model **Biology**: Population- dynamics; gene expression; cell cycle; natural selection; population size; immune system; basic reproduction- number;escherichia coli systems biology ndtabular	**Compute:** **Biology: ** Escherichia coli; gene expression; binding site; e coli; dna replication; binding sites; amino acids; rna polymerase; cell cycle; dna damage	**Compute:** Cloud computing; data analysis; data mining; data sets; machine learning; semantic web **biology:** Computational- biology;systems biology; gene expression; molecular biology

Topic4	Topic5	Topic6
**Compute**: **Biology**: Escherichia coli; e coli; amino acids; gene expression; saccharomyces cerevisiae; mass spectrometry; molecular weight; risk factorspublic health; heavy metals	**Compute**: **Biology:** Active site; escherichia coli; binding site; amino acids; circular dichroism; hydrogen bonds; hydrogen bond; cytochrome c; e coli; protein folding	**Compute**: **Biology:** Mass spectrometry; gene expression; breast cancer; cell lines; dna methylation; transcription- factors; liquid- chromatography; amino acids; oxidative stress; protein spots

Topic7	Topic8	Topic9
**Compute:** **Biology:** Amino acids; gene expression; x chromosome; skeletal muscle; reading frame; human genome; alternative splicing; breast cancer; linkage- disequilibrium; amino acid- sequence	**compute:** Data sets; machine learning; data set; data analysis **biology:** Gene expression; protein structure; supplementary- information; sequence alignment; protein sequences; dna sequences	**Compute:** Deep learning; wavelet transform; machine learning; data sets **Biology: ** Magnetic resonance- imaging; magnetic resonance white matter; image- reconstruction visual cortex; breast cancer

### Co-occurrence word network

To further investigate bioinformatics topics related to computational methods, we combine topic clustering and co-occurrence words analysis to construct a co-occurrence word network. The calculation method is as follows:

The data set of each topic was divided into six time slices to show the changes in each topic over time. From 1960 to 2019, each time slice represents 10 years. We calculated the tf-idf value of MESH phrases in different time slices in each topic, and then divided top 10 phrases into computational phrases and biology phrases according to MESH classification results. A co-occurrence word network between computational phrases and biology phrases is constructed to illustrate this time slice.

## Data Availability

Data were obtained from Microsoft Academic Graph (MAG) : https://www.microsoft.com/en-us/research/project/microsoft-academic-graph/

## Appendix 1: List of bioinformatics journals

1. BMC Bioinformatics
2. BMC Genomics
3. PLoS Biology
4. Genome Biology
5. PLoS Genetics
6. PLoS Computational Biology
7. BMC Research Notes
8. Bioinformatics
9. Molecular Systems Biology
10. BMC Systems Biology
11. Comparative and Functional Genomics
12. Bioinformation
13. Human Molecular Genetics
14. The Embo Journal
15. Cancer Informatics
16. Genome Medicine
17. Evolutionary Bioinformatics
18. Biochemistry
19. Algorithms for Molecular Biology
20. Eurasip Journal on Bioinformatics and Systems Biology
21. Journal of Molecular Biology
22. Molecular & Cellular Proteomics
23. Mammalian Genome
24. Source Code for Biology and Medicine
25. Biodata Mining
26. Journal of Computational Neuroscience
27. Journal of Proteome Research
28. Journal of Biomedical Semantics
29. Journal of Molecular Modeling
30. Bulletin of Mathematical Biology
31. Pharmacogenetics and Genomics
32. Statistical Methods in Medical Research
33. Neuroinformatics
34. Genomics
35. Protein Science
36. Physiological Genomics
37. Trends in Genetics
38. Journal of Proteomics
39. Proteomics
40. Trends in Biochemical Sciences
41. Journal of Biotechnology
42. trends in Biotechnology
43. Journal of theoretical Biology
44. Journal of Integrative Bioinformatics
45. Computational Biology and Chemistry
46. International Journal of Data Mining and Bioinformatics
47. MOJ Proteomics & Bioinformatics
48. Journal of Proteomics & Bioinformatics
49. Network Modeling Analysis in Health Informatics and Bioinformatics
50. The Open Bioinformatics Journal
51. Advances in Bioinformatics
52. Dictionary of Bioinformatics and Computational Biology
53. IEEE ACM Transactions on Computational Biology and Bioinformatics
54. Current Bioinformatics
55. Bioinformatics and Biology Insights
56. biometrics and Bioinformatics
57. Journal of Clinical Bioinformatics
58. International Journal of Bioinformatics Research and Applications
59. International Journal of Computational Biology and Drug Design
60. International Journal of Bioscience Biochemistry and Bioinformatics
61. Applied Bioinformatics
62. International Journal of knowledge Discovery in Bioinformatics
63. Mathematical Biology and Bioinformatics
64. Journal of Bioinformatics and Computational Biology
65. trends in Bioinformatics
66. Journal of Computational Biology
67. briefings in Bioinformatics
68. biotechnologia Journal of Biotechnology Computational Biology and Bionanotechnology
69. IPSJ Transactions on Bioinformatics
70. China Journal of Bioinformatics
71. Genomics Proteomics & Bioinformatics

## Appendix 2: List of bioinformatics conferences

1. APBC
2. BIBE
3. BIBM
4. B-interface
5. Biodevices
6. Bioinformatics
7. Biosig
8. Biosignals
9. Biostec
10. BMEI
11. BSBT
12. CIBCB
13. CMSB
14. CSB
15. DNA computing
16. ECCB
17. EuroGP
18. EvoBio
19. FOGA
20. GCB
21. GECCO
22. ISBI
23. ISBRA
24. ISMB
25. PRIB
26. PSB
27. RECOMB
28. WABI
29. WBIR
30. Brazilian symposium on bioinformatics
31. Data mining in bioinformatics
32. Computational intelligence methods for bioinformatics and biostatistics
33. Data and text mining in bioinformatics
34. International conference computational systems-biology and bioinformatics
35. Bioinformatics research and development
36. International conference on bioinformatics and biomedical engineering
37. International joint conferences on bioinformatics, systems biology and intelligent computing
38. International workshop on practical applications of computational biology and bioinformatics
39. International symposium health informatics and bioinformatics
40. Biocomputation, bioinformatics, and biomedical technologies
41. International conference bioscience, biochemistry and bioinformatics

## References

[1] Roos DS (2001). Bioinformatics-trying to swim in a sea of data. Science.

[2] Patra SK, Mishra S (2006). Bibliometric study of bioinformatics literature. Scientometrics.

[3] Chen Y-S, Leimkuhler FF (1986). A relationship between Lotka’s law, Bradford’s law, and Zipf’s law. J Am Soc Inf Sci.

[4] Glänzel W, Janssens F, Thijs B (2009). A comparative analysis of publication activity and citation impact based on the core literature in bioinformatics. Scientometrics.

[5] Song M, Kim S, Zhang G, Ding Y, Chambers T (2014). Productivity and influence in bioinformatics: a bibliometric analysis using pubmed central. J Am Soc Inf Sci.

[6] Song M, Kim SY (2013). Detecting the knowledge structure of bioinformatics by mining full-text collections. Scientometrics.

[7] Tang J, Zhang J, Yao L, Li J, Zhang L, Su Z. Arnetminer: extraction and mining of academic social networks. In: Proceedings of the 14th ACM SIGKDD international conference on knowledge discovery and data mining, 2008;990–998.

[8] Heo GE, Kang KY, Song M, Lee JH. Analyzing the field of bioinformatics with the multi-faceted topic modeling technique. BMC Bioinform 2017;18(Suppl 7). 10.1186/s12859-017-1640-x PMC5471940 28617229

[9] Le Q, Mikolov T. Distributed representations of sentences and documents. In: International conference on machine learning, 2014;1188–1196.

[10] Liu J, Tang T, Wang W, Xu B, Kong X, Xia F (2018). A survey of scholarly data visualization. IEEE Access.

[11] Gu D, Li J, Li X, Liang C (2017). Visualizing the knowledge structure and evolution of big data research in healthcare informatics. Int J Med Inform.

[12] Wu H, Wang M, Feng J, Pei Y. Research topic evolution in“bioinformatics”. In: 2010 4th international conference on bioinformatics and biomedical engineering, 2010;1–4.

[13] Song M, Yang CC, Tang X (2013). Detecting evolution of bioinformatics with a content and co-authorship analysis. Springerplus.

[14] Liao H, Tang M, Luo L, Li C, Chiclana F, Zeng XJ (2018). A bibliometric analysis and visualization of medical big data research. Sustainability (Switzerland).

[15] Havre S, Hetzler B, Nowell L. Themeriver: visualizing theme changes over time. In: IEEE symposium on information visualization 2000. INFOVIS 2000. Proceedings, 2000;115–123.

[16] Rafols I, Porter AL, Leydesdorff L (2010). Science overlay maps: a new tool for research policy and library management. J Am Soc Inform Sci Technol.

[17] Espadoto M, Martins RM, Kerren A, Hirata NS, Telea AC. Towards a quantitative survey of dimension reduction techniques. IEEE Trans Visual Comput Graph 2019. 10.1109/TVCG.2019.2944182 31567092

[18] Wang K, Shen Z, Huang C, Wu C-H, Dong Y, Kanakia A (2020). Microsoft academic graph: when experts are not enough. Quant Sci Stud.

[19] Song M, Heo GE, Kim SY (2014). Analyzing topic evolution in bioinformatics: investigation of dynamics of the field with conference data in DBLP. Scientometrics.

[20] Lau JH, Baldwin T. An empirical evaluation of doc2vec with practical insights into document embedding generation 2016. arXiv preprint arXiv:1607.05368

[21] McInnes L, Healy J, Astels S (2017). hdbscan: Hierarchical density based clustering. J Open Source Softw.

[22] McInnes L, Healy J, Melville J. Umap: Uniform manifold approximation and projection for dimension reduction 2018. arXiv preprint arXiv:1802.03426

